# The American Medical Association and New York’s New Code

**Published:** 1882-07

**Authors:** 


### THE AMERICAN MEDICAL ASSOCIATION AND NEW YORK'S NEW CODE.

It will be observed by reference to the proceedings of the American Medical Association, printed elsewhere, that this body, with gratifying unanimity and with a vim delightfully suggestive of intense earnestness, rejected the delegates from the New York State Medical Society on account of the code of ethics recently adopted by the society, which contained provisions at variance with the requirements of the code of the National Association.

The permanent members of the Association who are also members of the state society were not disturbed in their membership. A year of grace, it was understood, would be allowed, in which they could consider the matter.

We doubt not reflection will result in reconsideration of the whole question, the former action of the society will be wisely rescinded, and those members who progressed so impulsively will be invited to take back seats or else permitted to withdraw and to put into practice their pet theories, by an unrestrained association with the mongrel crew for which they profess such tender solicitude and such high regard.

The action of the Association will be accepted by every lover of professional good order and by every advocate of an uncompromising ethical standard with feelings of grateful satisfaction. It will commend anew this organization to the confidence and affection of the profession, and will establish it unalterably as the great and trusted conservator of American medical interests.

